# Prevalence of idiopathic pulmonary fibrosis in Japan based on a claims database analysis

**DOI:** 10.1186/s12931-022-01938-6

**Published:** 2022-02-08

**Authors:** Yasuhiro Kondoh, Takafumi Suda, Yoshie Hongo, Manami Yoshida, Shinzo Hiroi, Kosuke Iwasaki, Tomomi Takeshima, Sakae Homma

**Affiliations:** 1 Department of Respiratory Medicine and Allergy, Tosei General Hospital, Aichi, Japan; 2 The Second Division, Department of Internal Medicine, Hamamatsu University School of Medicine, Shizuoka, Japan; 3 Medical Affairs, Shionogi & Co., Ltd., 7F, Tekko Building, 1-8-2, Marunouchi, Chiyoda-ku, Tokyo, 100-0005 Japan; 4 Milliman, Inc., Tokyo, Japan; 5 Department of Advanced and Integrated Interstitial Lung Diseases Research, School of Medicine, Toho University, Tokyo, Japan

**Keywords:** Claims data, Epidemiology, Idiopathic pulmonary fibrosis, Japan, Prevalence

## Abstract

**Background:**

Idiopathic pulmonary fibrosis (IPF) is a cryptogenic chronic interstitial pneumonia with progressive fibrosis and a poor prognosis. A substantial number of epidemiological studies have been conducted in Europe and the United States (US). In contrast, in Japan, only one study reported the prevalence of IPF (10.0 per 100,000 population) using clinical data (2003–2007) from one prefecture; thus, the nationwide prevalence of IPF remains unknown. This study aimed to estimate the nationwide prevalence of IPF in Japan using a nationwide claims database.

**Methods:**

We extracted data from a Japanese claims database provided by Medical Data Vision (MDV database, April 2008–March 2019) containing data from approximately 28 million patients from 385 acute-care hospitals. Patients with IPF (those diagnosed with IPF at least once) from April 2017 to March 2018 were identified in the MDV database. The number of patients in the MDV database was extrapolated nationwide using the fourth NDB Open Data (April 2017–March 2018), and the prevalence was estimated using demographic data as denominators. The prevalence in the US, considering the same definition of IPF, was also calculated and compared with that in Japan.

**Result:**

The number of patients with IPF in the MDV database was 4278. The estimated nationwide number of patients in Japan was estimated to be 34,040 (mean age: 73 years, percentage of men: 73%), and the prevalence was 27 per 100,000 population. In comparison with that in the US, the prevalence was similar in men and relatively lower in women until the age of 75–79 years, and it was notably lower in both sexes aged ≥ 80 years.

**Conclusions:**

We report the nationwide IPF prevalence in Japan using data from claims databases for the first time. The prevalence estimated in this study was higher than that reported in a previous study. The difference might be due to differences in study settings and definitions of IPF. Further research should be performed to determine the prevalence more accurately and compare it with those in other countries.

**Supplementary Information:**

The online version contains supplementary material available at 10.1186/s12931-022-01938-6.

## Background

Idiopathic pulmonary fibrosis (IPF) is a cryptogenic chronic interstitial pneumonia with progressive fibrosis and poor prognosis that mainly develops in older people. Primary symptoms at the onset of the disease are dry cough and exertional dyspnea [1–4]. The median patient survival time is reportedly 2–5 years after initial diagnosis [5–8]. International standards for the diagnosis and treatment of IPF are outlined in the guidelines generated in 2011 and revised in 2015 and 2018 by The American Thoracic Society, The European Respiratory Society, The Japanese Respiratory Society, and The Latin American Thoracic Association [1, 9, 10].

However, the nationwide prevalence of IPF is still unknown in Japan. While a substantial number of epidemiological studies have been conducted in Europe and the United States (US), only two studies, to the best of our knowledge, provide information about the prevalence of IPF in Japan. IPF is considered an incurable disease, and medical expenses are partially supported to certified patients with incurable diseases, including IPF, by the government in Japan. One study investigating the nationwide prevalence of idiopathic interstitial pneumonia (IIP) in 2005 targeted only patients who received certifications and had moderate or severe disease severity [11]. Therefore, the estimated prevalence of IIP, at 3.44 per 100,000 population, 85.7% of whom had IPF, might be underestimated. Another study reported the prevalence of IPF to be 10.0 per 100,000 population using clinical data from 2003 to 2007; the prevalence was calculated among patients with Certified Medical Benefits in Hokkaido prefecture. Although the study included patients who were diagnosed with IPF regardless of severity, the study was conducted in only a prefecture and not nationwide [8]. To date, the latter prevalence has been considered to be representative of the nationwide prevalence [12] because of the study population total and the background of the population. Many ancestors originated from various areas nationwide and migrated to this area; thus, the genetic background of the population in Hokkaido prefecture is thought to be representative of the genetic background of the entire population. However, the former study reported that the prevalence ranged from < 2.0 to ≥ 4.0 per 100,000 person among prefectures [11], suggesting that the prevalence should be calculated based on nationwide data. Moreover, the prevalence of IPF in Japan is considered to be lower than that in Western countries, which is reportedly 14.0–27.9 per 100,000 population [13, 14]. The lower prevalence in Japan on the basis of previous studies may be attributed to differences in countries or races as well as differences in investigation and estimation methods. Information about the nationwide prevalence of IPF in Japan is required to understand the status of IPF and differences among countries and races.

The aim of this study was to estimate the nationwide prevalence of IPF in Japan using nationwide claims databases. Additionally, the prevalence of IPF in the US was calculated using the same method and compared with that in Japan.

## Methods

### Study design and data sources

This claims-based study estimated the prevalence of IPF in Japan and the US using two claims databases for each country.

One of the Japanese databases is maintained by Medical Data Vision Co., Ltd. (hereafter the MDV database; April 2008–March 2019), which contains the claims data of approximately 28 million patients from 385 diagnosis procedure combination-designated acute care hospitals, termed DPC hospitals (as of August 2019). The MDV database included the data of all patients visiting the corresponding hospitals regardless of the patients’ age and type of health insurance. Because the database did not contain population data and thus the prevalence could not be estimated solely from these data, we used two additional data sources. We used NDB Open Data (fourth NDB Open Data: April 2017–March 2018) [15] to estimate the nationwide number of patients and used demographic data [16] as denominators. NDB Open Data is a publicly available summary spreadsheet from the National Database of Health Insurance Claims and Specific Health Checkups of Japan (NDB) published by the Ministry of Health, Labour and Welfare. The NDB contains almost all health insurance claims data and specific health checkup data associated with the national health insurance system. In the NDB Open Data, 100 of the most prescribed drugs for each therapeutic category in a 1-year period from April are summarized for each age and sex group. Pirfenidone, which is one of two antifibrotic drugs (along with nintedanib) recommended as first-line treatments in the guidelines [1, 9], is included in the fourth NDB Open Data.

Another Japanese claims database, provided by JMDC Inc. (hereafter the JMDC database; January 2015–June 2019), contains data from company employees and their families subscribing to the corresponding health insurance societies. The database includes all medical records regardless of the type of healthcare facility in which medical care was provided. The database includes population data, which allowed us to calculate the prevalence. However, few data were available for individuals aged ≥ 65 years, and almost no data were available for those aged ≥ 75 years in the database. Therefore, we used the JMDC database to calculate the prevalence of IPF for those aged < 65 years to assess the validity of the results from the MDV database.

To estimate the US prevalence, the IBM MarketScan Commercial Database provided by IBM Corporation (hereafter the MarketScan database; 2017) and Medicare 5% sample data provided by Centers for Medicare & Medicaid Services (hereafter the Medicare database; 2018) were used. The MarketScan database consists of data from commercial health insurance companies, including population data. In the database, data for those aged ≥ 65 years were limited because people usually change their health insurance to Medicare, a national health insurance program, in the US. Thus, it was used to calculate the prevalence for those aged < 65 years. The Medicare 5% dataset contains a sample of claims data, including population data, for those with government-provided health insurance in the US. This source was used to calculate the prevalence among those aged ≥ 65 years.

### Analysis

Patients diagnosed with IPF at least once were defined as patients with IPF. The diagnosis of IPF was identified by the disease name idiopathic pulmonary fibrosis in the Japanese databases and by the International Classification of Diseases, 10th Revision (ICD-10) code J84.112 (idiopathic pulmonary fibrosis) in the US databases. Data from a 1-year period, from April 2017 through March 2018 for the MDV database, from January through December 2017 for the MarketScan database, and from January through December 2018 for the JMDC database and Medicare database, were used to estimate the prevalence rates.

**Fig. 1:**
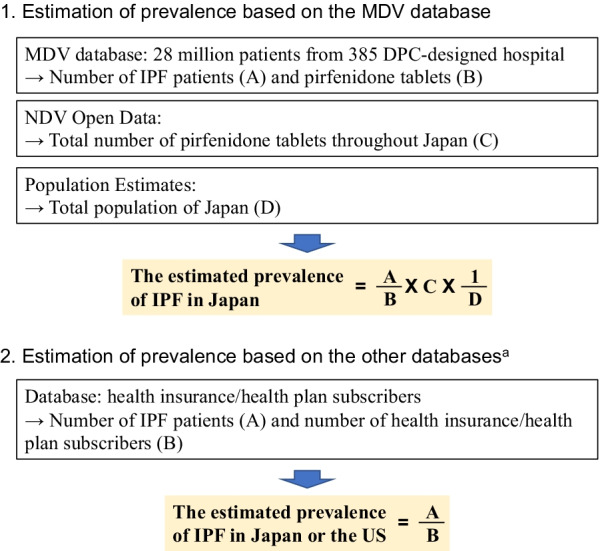
Diagram of the prevalence estimation method. ^a^The other databases includes the database provided by JMDC, IBM MarketScan Commercial Database, and Medicare 5% sample data. MDV database consists of claims data from acute care hospitals, and does not contain population data. NDB Open Data is a publicly available summary spreadsheet from National Database of Health Insurance Claims and Specific Health Checkups of Japan (NDB), which contains almost all health insurance claims data. In the NDB Open Data, 100 of the most prescribed drugs for each therapeutic category, including pirfenidone, in a one-year period from April are summarized for each age and sex group. *IPF* Idiopathic pulmonary fibrosis; *MDV* Medical Data Vision

The prevalence on the basis of the MDV database was calculated by extrapolating the number of patients in the MDV database using a ratio of patients to pirfenidone tablets calculated from the MDV database and the number of prescriptions of pirfenidone tablets in NDB Open Data as follows: (1) ratio of patients to pirfenidone tablets = (total number of patients with IPF in the MDV database)/(total number of prescriptions of pirfenidone tablets in the MDV database); (2) nationwide number of patients with IPF = (ratio of patients to pirfenidone tablets) × (number of prescriptions of pirfenidone tablets from NDB Open Data); and (3) prevalence = (nationwide number of patients with IPF)/(Japanese population) (Fig. 1). Notably, the ratio of patients to pirfenidone tablets was calculated to extrapolate the number of patients with IPF in the MDV database to the number of patients nationwide; thus, the patients from the MDV database were all patients with IPF and were not limited to those who received pirfenidone tablets. The prevalence was also calculated according to age group (5-year intervals) and sex. The prevalence using the other claims databases was calculated by dividing the number of patients with IPF by the number of subscribers in each age and sex group in each database (Fig. 1). To assess the estimated prevalence, the prevalence by year (from 2015 to 2019) from the JMDC database and the prevalence by race using the Medicare database, as well as the ratio of patients to pirfenidone tablets from the JMDC database, were also calculated.

The estimated prevalence in Japan and the US by age and sex groups were displayed descriptively in the same graph, with 95% confidence intervals, and compared to observe the difference between the countries.

Further, the percentage of patients who had undergone any of the examinations was calculated for patients ≥ 65 years to assess differences in diagnosis characteristics between the countries using the MDV and Medicare databases. Of the patients diagnosed with IPF in 2018, the percentages of those who underwent high-resolution computed tomography, bronchoalveolar lavage, transbronchial lung biopsy, or surgical lung biopsy at least once during the calendar year 2018 were calculated.

We used SAS ver. 9.4 (SAS Institute, Cary, NC, USA) and Microsoft Excel 2016 (Microsoft, Redmond, WA, USA) for the analyses.

## Results

### Estimated prevalence of IPF in Japan

In the MDV database, we identified 4278 patients with IPF: 1067 patients with prescription of pirfenidone from April 2017 to March 2018 (1033 patients with diagnosis of IPF within the same period, and 34 patients with diagnosis only outside of the period) and 3211 patients without prescription of pirfenidone and with diagnosis of IPF within the period. For the patients, 1,206,310 prescriptions of pirfenidone tablets (1,182,597 and 23,713 tablets for those who were diagnosed within the period and outside of the period, respectively) were identified, and the ratio of patients to pirfenidone tablets was 0.00355. The ratios in each age and sex group were mostly similar (Additional file 1: Fig. S1), so the same value was applied to all age and sex groups. The estimated nationwide number of patients was calculated to be 34,040 (mean age: 73 years, percentage of men: 73%), and the prevalence was 27 per 100,000 population. The prevalence was higher in men than in women, and the prevalence in men ≥ 65 years was 3.39-fold higher than that in women (Additional file 1: Fig. S2a). The prevalence was approximately 7.3 and 4.0 per 100,000 population in men and women aged 50–54 years and increased with age to 48 and 15 per 100,000 population aged 60–64 years, respectively (Additional file 1: Fig. S2a). In both sexes, the peak prevalence was observed in those aged 75–79 years (Additional file 1: Fig. S2a).

The number of members in the JMDC database in 2018 (see Additional file 1: Fig. S3 for age- and sex-specific populations) was 5.2 million, and the number of patients with IPF was 217. The prevalence was approximately 3.5 and 2.4 per 100,000 population in men and women aged 50–54 years and increased with age to 39 and 9.3 per 100,000 population in men and women aged 60–64 years, respectively (Additional file 1: Fig. S2b). The age- and sex-specific prevalence was confirmed to be similar from 2015 through 2019 (Additional file 1: Fig. S4). The ratio of patients to pirfenidone tablets was 0.00238.

### Estimated prevalence of IPF in the US

The population in the MarketScan database was 26.3 million, with a small number of individuals ≥ 65 years (see Additional file 1: Fig. S5 for age- and sex-specific populations). The number of patients with IPF was 1769.

The Medicare database included 3.1 million individuals, with the majority comprising those aged 65–69 years; the number of individuals decreased with age (see Additional file 1: Fig. S6 for age- and sex-specific populations). In total, 3486 patients with IPF were identified.

**Fig. 2:**
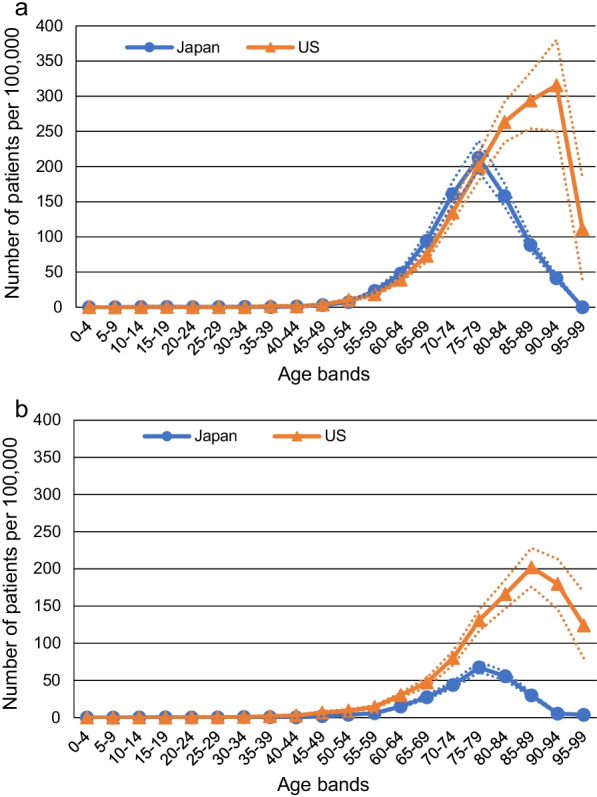
Age- and sex-specific prevalence of idiopathic pulmonary fibrosis in Japan and the US in **a** men and **b** women. The prevalence was estimated based on the Medical Data Vision Co., Ltd database, fourth NDB Open Data [15], and demographic data [16] (April 2017–March 2018) for Japan and the IBM MarketScan Commercial Database (January–December 2017) for aged < 65 years and Medicare 5% sample data (January–December 2018) for aged ≥ 65 years for the US. Dotted lines indicate 95% confidence intervals

The prevalence was calculated from the databases according to age group and sex, as shown in Additional file 1: Fig. S7a (MarketScan database) and Additional file 1: Fig. S7b (Medicare database). We combined the prevalence of IPF among those aged < 65 years in the MarketScan database and those aged ≥ 65 years in the Medicare database on the graph (Additional file 1: Fig. S8), and the combined data seemed to show a continuous pattern in both men and women. Therefore, we determined the age- and sex-specific prevalence rates in the US using the results from these databases (Fig. 2). The prevalence was higher in men and 1.47-fold higher in men aged ≥ 65 years than in women. The prevalence generally increased with age to the highest rates, which were observed in men aged 90–94 years (Fig. 2a) and women aged 85–89 years (Fig. 2b).

We compared the prevalence by race in those aged ≥ 65 years. The prevalence tended to be lower in black individuals in most age and sex groups, and no particular difference was observed among other races (Additional file 1: Fig. S9).

### Difference between the prevalence in Japan and the US

The prevalence was similar or slightly higher in Japan (from the MDV database) than in the US until the ages of 75–79 years in men (Fig. 2a). However, the prevalence decreased with age ≥ 80 years in Japan, while it continued to increase with age in the US until the age of 90–94 years. In women, the prevalence was lower in Japan than in the US, with an increase in the difference with age (Fig. 2b). Similar to that in men, the peak prevalence was observed in those aged 75–79 years in Japan, while it increased until the age of 85–89 years in the US.

To explore the reasons for the difference in prevalence, particularly in those aged ≥ 80 years, between the countries, we compared the prevalence in Japan to that among Asian subgroups in the US (Additional file 1: Fig. S10). The prevalence among Asian subgroups was higher than those in most age groups and in both sexes in Japan.

### Percentage of IPF patients who underwent examinations

**Fig. 3:**
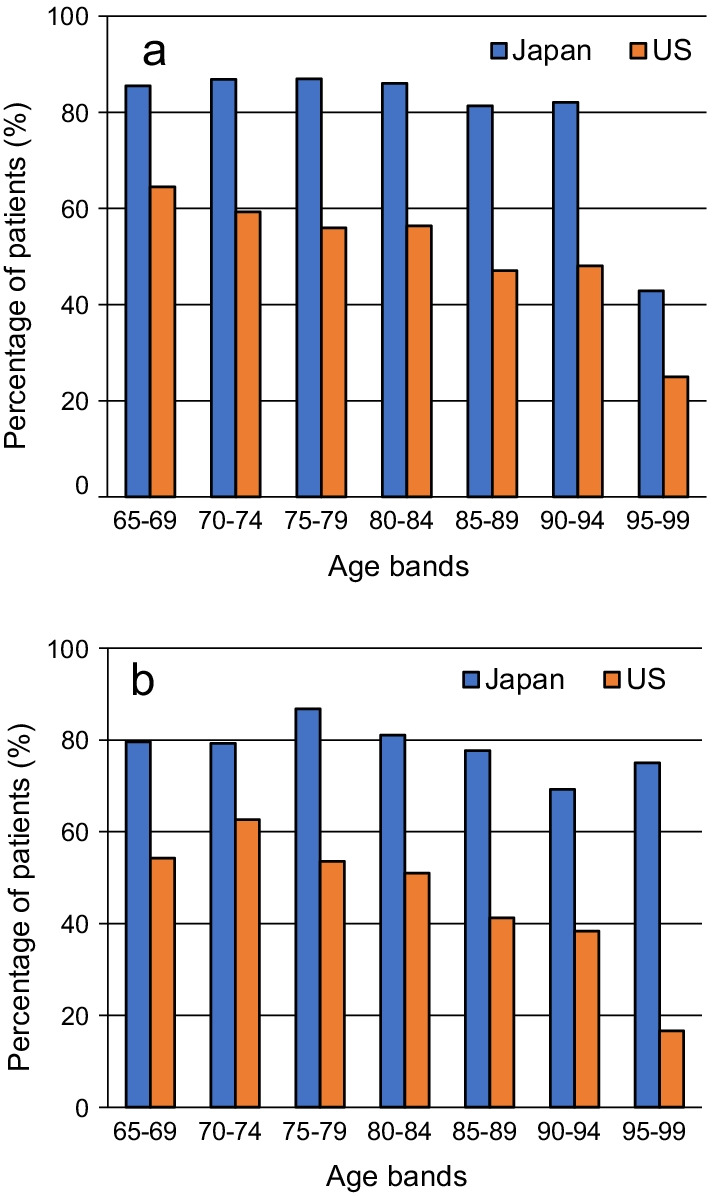
Percentage of patients with idiopathic pulmonary fibrosis with examinations for those aged ≥ 65 years in Japan and the US in **a** men and **b** women. The percentage was calculated by dividing the number of patients who underwent examinations at least once during the 2018 calendar year by the number of patients diagnosed with idiopathic pulmonary fibrosis in 2018. Examinations included high-resolution computerized tomography, bronchoalveolar lavage, transbronchial lung biopsy, or surgical lung biopsy

The percentage of IPF patients who had undergone any of the specified examinations at least once was more than 80% by the age of 90–94 years in men and approximately 80% by the age of 85–89 years and 70% by the age of 95–99 years in women in Japan (Fig. 3). The percentage was approximately 60% or lower in all age and sex groups and < 50% in those aged ≥ 85 years in the US.

## Discussion

This study estimated the nationwide prevalence of IPF in Japan by developing a method combining data from multiple sources. To the best of our knowledge, this is the first study to investigate the nationwide prevalence of IPF in Japan. The estimated prevalence was 27 per 100,000 population. The age group with the highest prevalence was 75–79 years in both sexes. In comparison with that in the US, the prevalence was similar in men and relatively lower in women until the age of 75–79 years, while it was notably lower in both sexes aged ≥ 80 years.

A large-scale claims database comprising comprehensive data from individuals regardless of the severity of the disease could be expected to be a reliable data source to estimate nationwide prevalence. The MDV database and JMDC database are the major claims databases available in Japan. Because of a lack of data from older patients in the JMDC database, the MDV database was used to estimate the nationwide prevalence, and the JMDC database was used to assess the effect due to the characteristics of the MDV data and the methodology. To expand the number of patients in the MDV database to achieve a nationally representative sample, we calculated the ratio of patients to pirfenidone tablets in the MDV database and applied it to the number of prescriptions of pirfenidone tablets in NDB Open Data. The ratio of patients to pirfenidone tablets in the MDV database (0.00355) was larger than that in the JMDC database (0.00238). This result suggests that the patients in the MDV database might have received fewer treatments with pirfenidone even though they had attended large hospitals. A possible reason is that if a patient visited both DPC and non-DPC hospitals and the person received a prescription of pirfenidone at only the non-DPC hospital, the prescription was recorded in the JMDC database but not in the MDV database. Consequently, the ratio in the MDV database might be high, causing overestimation of the prevalence.

The prevalence in Japan was estimated to be 27 per 100,000 population in this study. It was higher than that estimated in a previous study in Hokkaido prefecture, at 10.0 per 100,000 population [8]. We deduce three potential reasons for the gap between them. The first reason is due to the definition of the patients. In a previous study, patients with IPF were identified on the basis of clinical records, high-resolution computed tomography findings, and the results of pathologic diagnosis (when a surgical biopsy was performed). This stricter definition compared with that used in the present study could contribute to the lower prevalence. The effect of the difference in the definition of IPF on the prevalence was previously reported in the US. The prevalence using ‘narrow definition,’ requiring specific examination findings, was less than half of that calculated considering the ‘broad definition:’ at least one diagnosis of IPF [14]. Although the percentage of patients who underwent an examination was relatively high in Japan, the prevalence in the present study might be overestimated. The second possible reason is associated with the differences in geographic areas. This does not clarify why our estimated rate was higher than that in the previous study limited for Hokkaido prefecture; however, it is possible that the difference in the scale of the target area contributed to the differential results. Finally, the target period of investigation could contribute to the difference. The previous study estimated the prevalence based on data from 2003 to 2007, while the period in this study was from 2017 to 2018. IPF is a relatively uncommon disease, and the definition of the disease was not widely recognized in or before 2003 [12]. Recognition of the disease has increased after international guidelines and national guidelines were formulated and revised in 2011 [1], 2015 [9], 2017 [17], and 2018 [10], and antifibrotic drugs as therapeutic medications were released on the market in 2008 and 2015. Consequently, the number of diagnoses might have increased, contributing to the increase in prevalence.

The prevalence in the US was consistent with that in a previous study (with a definition similar to that in our study): 192.0 per 100,000 population and 227.2 per 100,000 population [13], respectively, in those aged ≥ 75 years. When comparing Japan and the US in this study, the prevalence in the age groups ≥ 80 years was notably lower in Japan than in the US. We considered that the reason for the difference is that older patients in Japan received an IPF diagnosis less frequently than those in the US. In Japan, many older patients tend not to receive antifibrotic drugs because of concerns about side effects due to their age and careful consideration of the time to start treatment. A survey result indicated that there are more doctors who do not administer treatment at the early stage of the disease in Japan than in Europe and Canada [18]. As a diagnosis without associated treatment may not be recorded in the Japanese medical fee payment system, patients might be diagnosed with other diseases, such as interstitial pneumonia or idiopathic interstitial pneumonia, unless they receive antifibrotic drugs. Thus, if older patients in Japan received antifibrotic treatment less frequently than those in the US, it is likely that fewer Japanese patients received a diagnosis of IPF, resulting in underestimation of the number of older patients in Japan. In addition, the number of patients who are suspected of having IPF but do not undergo further investigation (e.g. if a patient was elderly and not treated and managed by a family physician, suspected IPF might not be investigated) might be higher in Japan than in the US. As our study was based on the record of diagnosis, we could not include patients who were not investigated in the hospital. We also speculated that this difference may be associated with differences in diagnostic methods between the countries, so we calculated the percentage of patients who had undergone such examinations. As a result, the percentage was higher in Japan than in the US (Fig. 3), suggesting that different diagnostic methods may contribute to the difference in the prevalence between the countries. In addition to the aforementioned reasons, the following possible reasons could exist, although future investigation is imperative. First, differences in lifestyles and race might have caused the difference in prevalence. An association of some environmental factors, occupational history, and smoking habit with IPF risk has been indicated, although the differences between countries are unknown [19]. A review article suggested the possibility that some clinical aspects, such as the cause of death and prognostic factors, of patients with IPF may be different between patients in Japan and those in other countries, and the authors hypothesized that ethnic differences contributed to the difference in prevalence [20]. However, our analysis did not show a clear difference in prevalence between Asian and other races in the US. Second, recognition of IPF might be lower among Japanese healthcare professionals, particularly among nonspecialists.

The higher male to female prevalence ratio in Japan (3.39) than in the US (1.46) was consistent with those in previous studies [8, 14]. We could not identify the exact reason for this difference, but differences in smoking rates could contribute to the difference in the ratio between the countries. Smoking is reported to be associated with an increased risk for IPF [21], and the difference in smoking rates between men and women was larger in Japan (smoking rate: 33.2% in men and 10.5% in women) than in the US (30.9% and 19.3%) in those aged ≥ 15 years in 2018 [22].

## Limitations

Some limitations should be considered in this study. We identified patients with IPF based on a diagnosis of IPF. Thus, patients without a diagnosis were not included even if they developed IPF. Since there were no chart data for individual patients in this database, the accuracy of the diagnoses of IPF could not be evaluated. In addition, as the diagnosis of IPF was made in each hospital, it is not clear whether the diagnosis was adequately made, whether the diagnosis was determined based on a multidisciplinary approach, or to what extent the multidisciplinary approach was followed when the diagnosis was made. We assumed that the ratio (IPF patients/pirfenidone tablets) in the MDV database was equal to that in NDB Open Data when estimating the nationwide prevalence in Japan. However, this assumption might not be exactly appropriate. Moreover, although pirfenidone is indicated only for IPF, it may be prescribed for patients other than those with a definitive IPF diagnosis. If this is true, then the nationwide number of patients with IPF estimated based on the total prescription number of pirfenidone tablets in NDB Open Data was overestimated. Finally, although we compared the prevalence in Japan with that in the US based on the same definition, the prevalence might not reflect the actual prevalence considering that previous studies reported the prevalence using both the ‘narrow definition’ and ‘broad definition.’ Further studies on diagnosis differences should be performed to assess differences among countries.

## Conclusions

This study reported the nationwide IPF prevalence in Japan using data from claims databases. The prevalence in Japan was estimated to be 27 per 100,000 population, which was higher than that reported in previous studies. The difference might be due to differences in the definitions of IPF and the settings of the studies. The prevalence in Japan was lower than that in the US, particularly in patients aged ≥ 80 years. Further research should be performed to determine the prevalence more accurately and compare it with that in other countries.

## Supplementary Information

**Additional file 1: Figure S1.** Age- and sex-specific ratios of patients with idiopathic pulmonary fibrosis to pirfenidone tablets in the MDV database (April 2017–March 2018). **Figure S2.** Age- and sex-specific prevalence of idiopathic pulmonary fibrosis in Japan using the (a) MDV database (April 2017–March 2018) and (b) JMDC database (January–December 2018). The fourth NDB Open Data and demographic data (April 2017–March 2018) were used with the MDV database to calculate the prevalence. **Figure S3.** Age- and sex-specific populations in the JMDC database (January–December 2018). **Figure S4.** Age- and sex-specific prevalence of idiopathic pulmonary fibrosis by year (2015–2019) in the JMDC database. **Figure S5.** Age- and sex-specific populations in the MarketScan database (January–December 2017). **Figure S6.** Age- and sex-specific populations in the Medicare database (January–December 2018). **Figure S7.** Age- and sex-specific prevalence of idiopathic pulmonary fibrosis in the US using the (a) MarketScan database (January–December 2017) and (b) Medicare database (January–December 2018). **Figure S8.** Age- and sex-specific prevalence of idiopathic pulmonary fibrosis in the MarketScan database (< 65 years, January–December 2017) and the Medicare database (≥ 65 years, January–December 2018). **Figure S9.** Race-, age- and sex-specific prevalence of idiopathic pulmonary fibrosis in the group aged ≥ 65 years in the Medicare database (January–December 2018). Prevalence is shown as the number per 100,000 population. Colors are assigned for the number of patients from low to high for each sex as indicated in the color scale bar. **Figure S10.** Age- and sex-specific prevalence of idiopathic pulmonary fibrosis in all races and the Asians subgroup in the US and Japan. The numbers in parentheses represent the numbers of patients with idiopathic pulmonary fibrosis in the Asian subgroups. The prevalence was estimated based on the Medical Data Vision Co., Ltd database, the fourth NDB Open Data, and demographic data (April 2017–March 2018) for Japan and the Medicare 5% sample data (January–December 2018) for the US.

## Data Availability

The claims data that support the findings of this study are available from Medical Data Vision Co., Ltd. for the MDV database, JMDC Inc. for the JMDC database, IBM Watson Health for the MarketScan database, and the Centers for Medicare and Medicaid Services for the Medicare database. Restrictions apply to the availability of these data, which were used under license for the current study, and so are not publicly available. Data are not available from the authors without permission from Medical Data Vision Co., Ltd., JMDC Inc., IBM Watson Health, and the Centers for Medicare and Medicaid Services.

## Abbreviations

ICD-10: International Classification of Diseases, 10th Revision
IIP: Idiopathic interstitial pneumonia
IPF: Idiopathic pulmonary fibrosis
MDV: Medical Data Vision Co., Ltd.
NDB: National Database of Health Insurance Claims and Specific Health Checkups of Japan
US: United States
