# Supplementary material for: Prevalence of idiopathic pulmonary fibrosis in Japan based on a claims database analysis
Source: Respir Res. 2022 Feb 8;23:24. doi: 10.1186/s12931-022-01938-6 (PMC8822670; doi:10.1186/s12931-022-01938-6)
Supplement: Supplementary file 1 — Additional file 1: Figure S1. Age- and sex-specific ratios of patients with idiopathic pulmonary fibrosis to pirfenidone tablets in the MDV database (April 2017–March 2018). Figure S2. Age- and sex-specific prevalence of idiopathic pulmonary fibrosis in Japan using the (a) MDV database (April 2017–March 2018) and (b) JMDC database (January–December 2018). The fourth NDB Open Data and demographic data (April 2017–March 2018) were used with the MDV database to calculate the prevalence. Figure S3. Age- and sex-specific populations in the JMDC database (January–December 2018). Figure S4. Age- and sex-specific prevalence of idiopathic pulmonary fibrosis by year (2015–2019) in the JMDC database. Figure S5. Age- and sex-specific populations in the MarketScan database (January–December 2017). Figure S6. Age- and sex-specific populations in the Medicare database (January–December 2018). Figure S7. Age- and sex-specific prevalence of idiopathic pulmonary fibrosis in the US using the (a) MarketScan database (January–December 2017) and (b) Medicare database (January–December 2018). Figure S8. Age- and sex-specific prevalence of idiopathic pulmonary fibrosis in the MarketScan database (< 65 years, January–December 2017) and the Medicare database (≥ 65 years, January–December 2018). Figure S9. Race-, age- and sex-specific prevalence of idiopathic pulmonary fibrosis in the group aged ≥ 65 years in the Medicare database (January–December 2018). Prevalence is shown as the number per 100,000 population. Colors are assigned for the number of patients from low to high for each sex as indicated in the color scale bar. Figure S10. Age- and sex-specific prevalence of idiopathic pulmonary fibrosis in all races and the Asians subgroup in the US and Japan. The numbers in parentheses represent the numbers of patients with idiopathic pulmonary fibrosis in the Asian subgroups. The prevalence was estimated based on the Medical Data Vision Co., Ltd database, the fourth NDB Open Data, and demograp [file 12931_2022_1938_MOESM1_ESM.pdf]

Additional File 1

Prevalence of idiopathic pulmonary fibrosis in Japan based on a claims database analysis

Fig. S1.

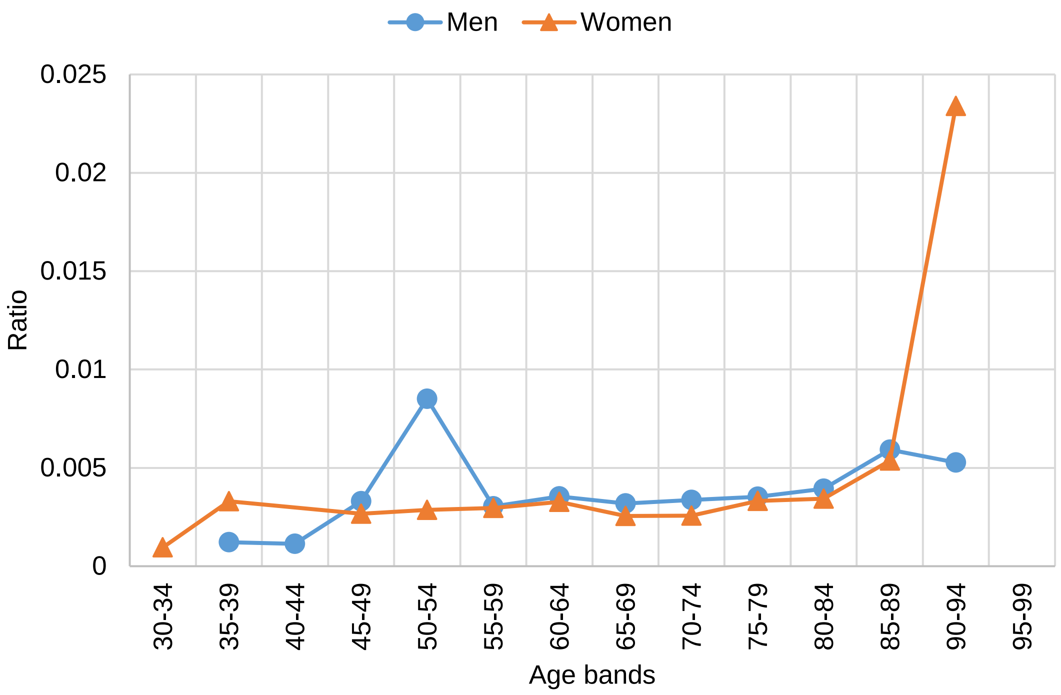

Age- and sex-specific ratios of patients with idiopathic pulmonary fibrosis to pirfenidone tablets in the MDV database (April 2017–March 2018). MDV, Medical Data Vision.

Fig. S2.

a

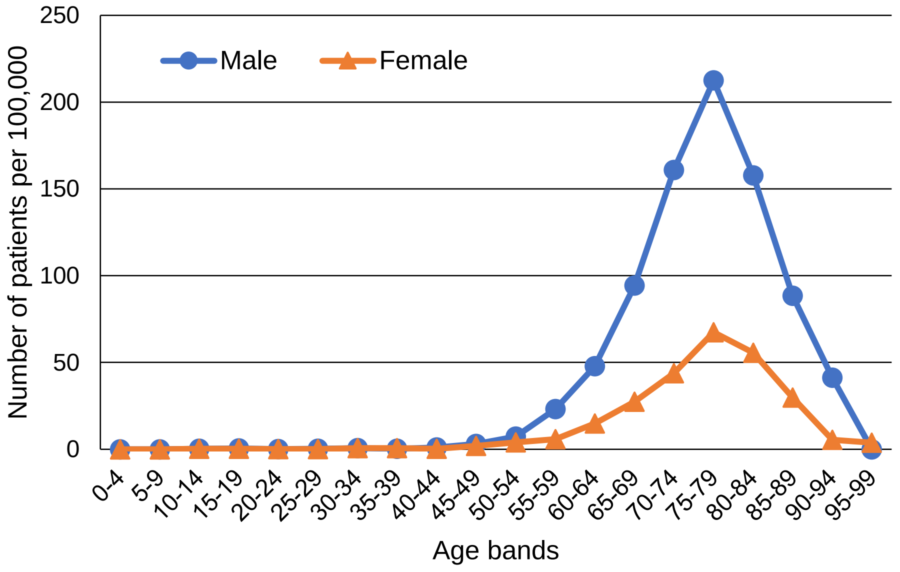

b

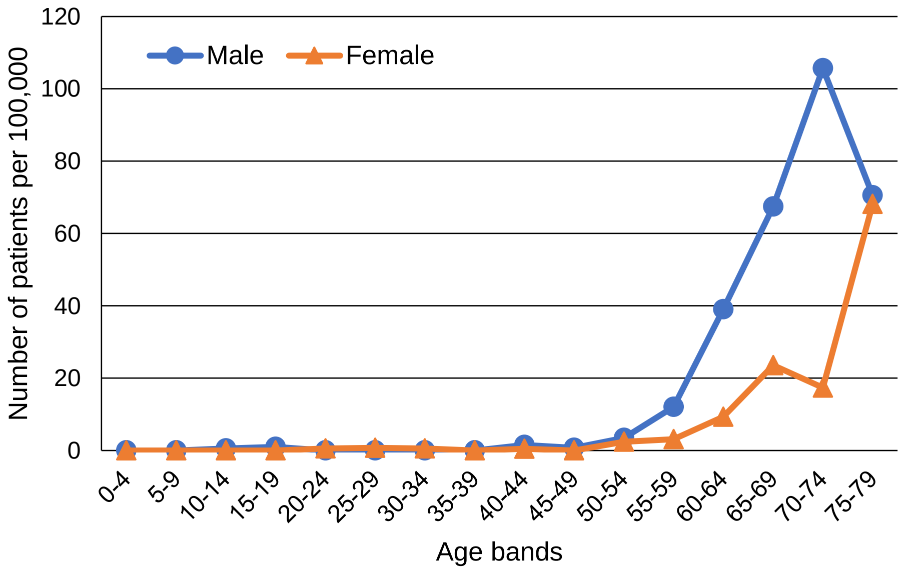

Age- and sex-specific prevalence of idiopathic pulmonary fibrosis in Japan using the (a) MDV database (April 2017–March 2018) and (b) JMDC database (January–December 2018). Fourth NDB Open Data and demographic data (April 2017–March 2018) were also used with the MDV database to calculate the prevalence. MDV, Medical Data Vision.

Fig. S3.

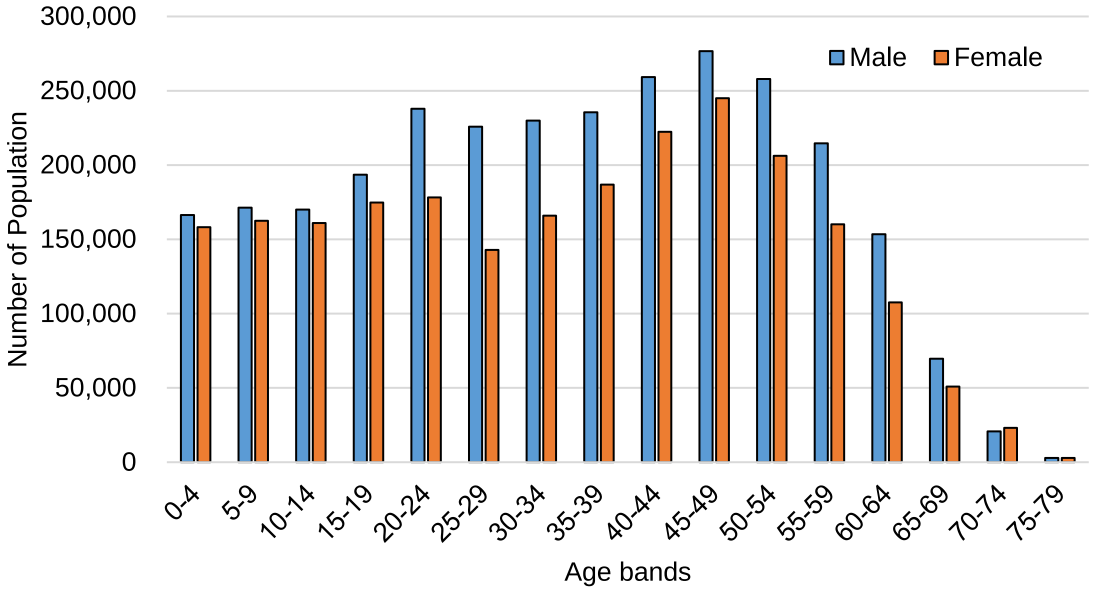

Age- and sex-specific populations in the JMDC database (January–December 2018).

Fig. S4.

a. Male

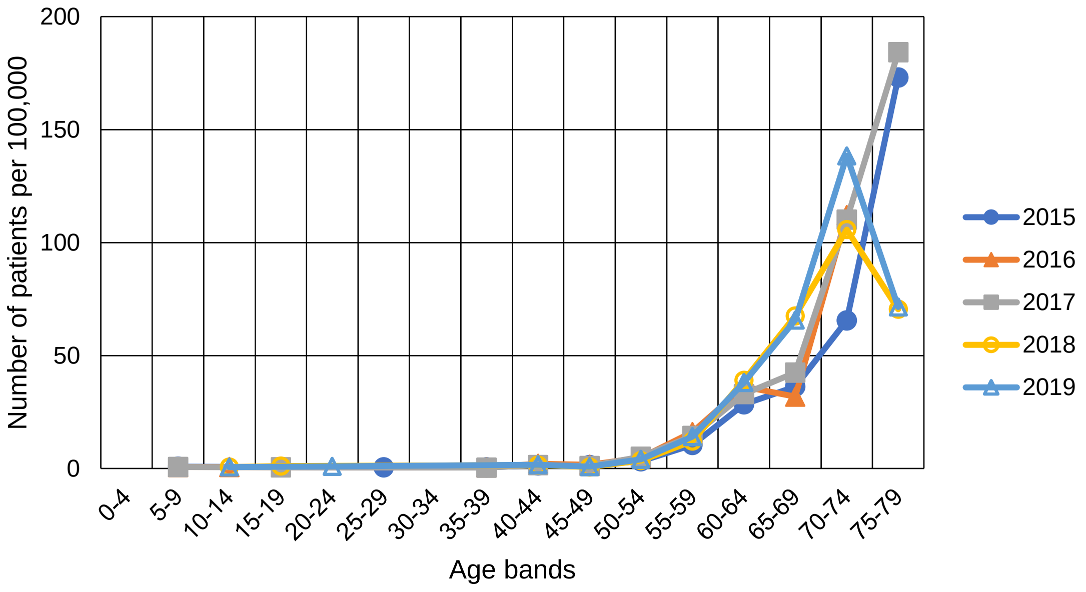

b. Female

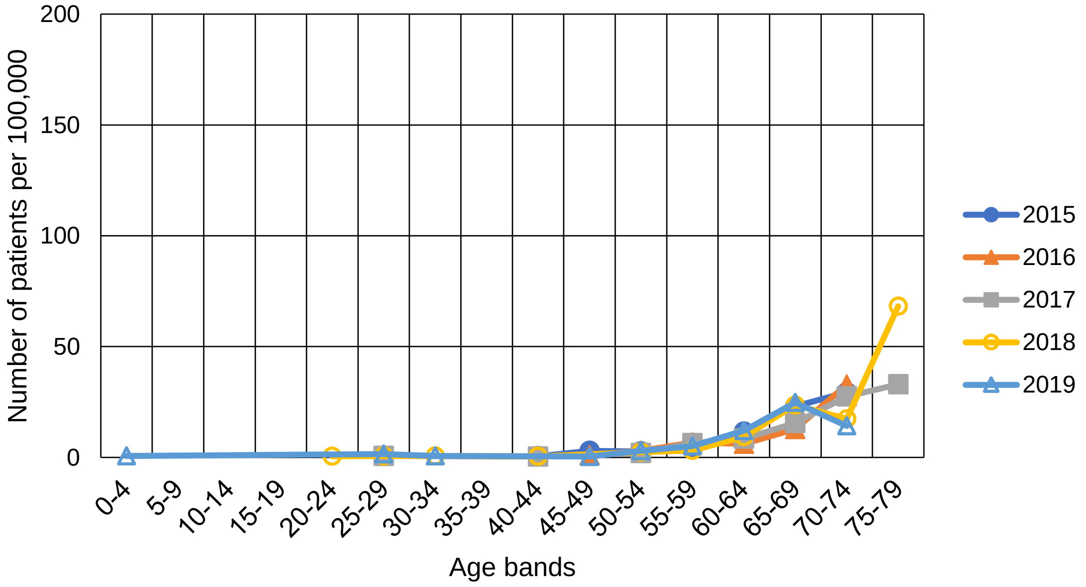

Age- and sex-specific prevalence of idiopathic pulmonary fibrosis by year (2015–2019) in the JMDC database.

Fig. S5.

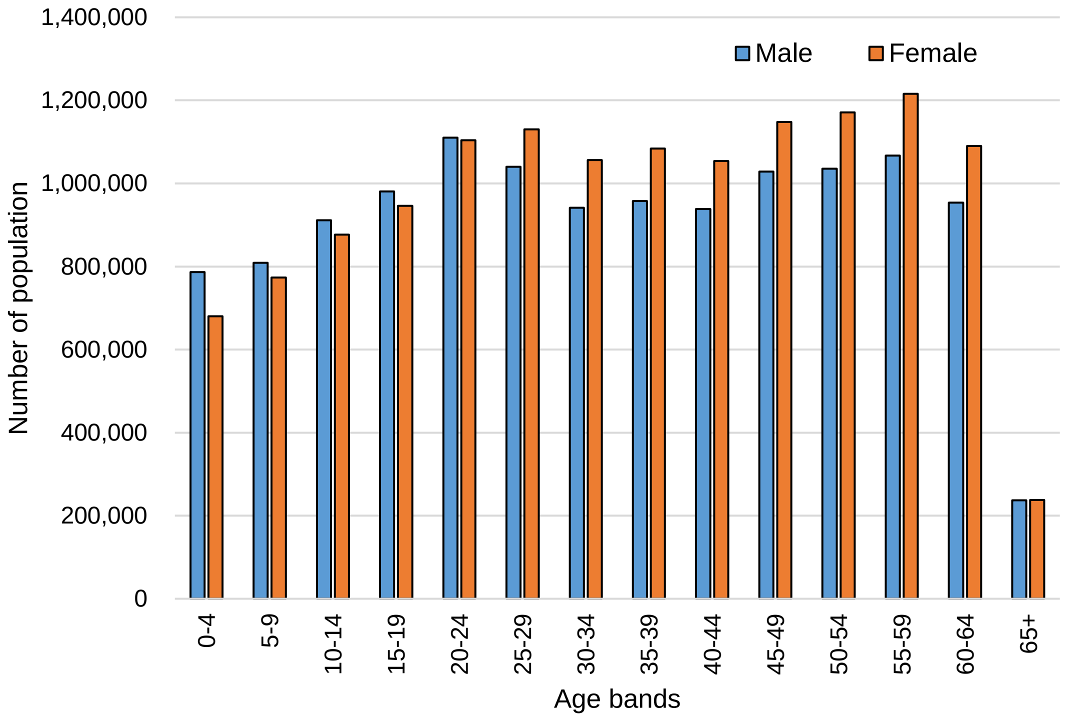

Age- and sex-specific populations in the MarketScan database (January–December 2017). MarketScan database, IBM MarketScan Commercial Database.

Fig. S6.

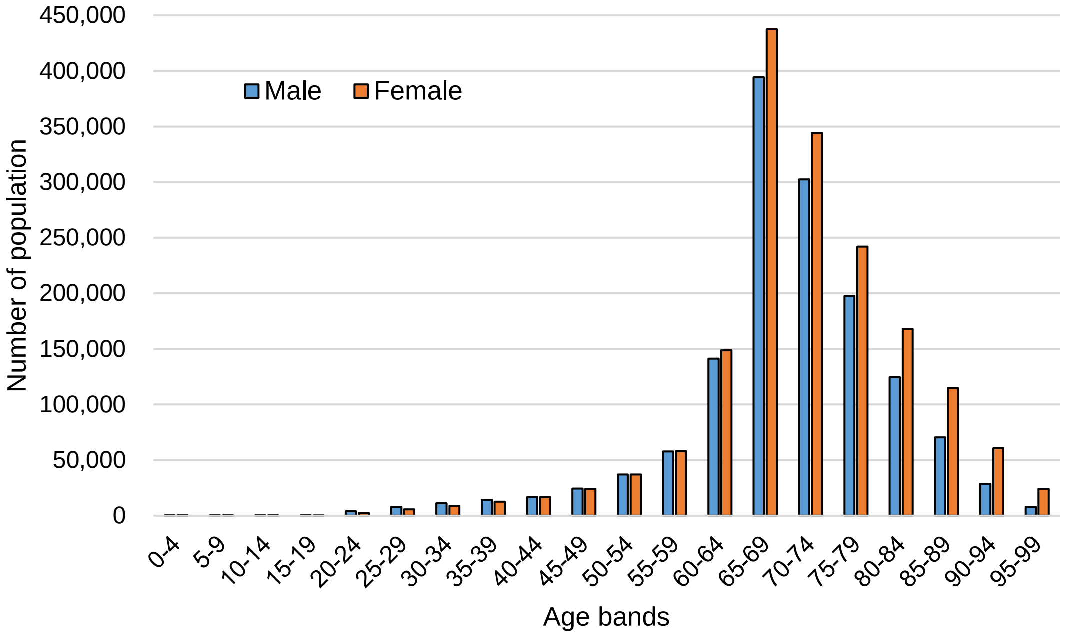

Age- and sex-specific populations in the Medicare database (January–December 2018). Medicare database, Medicare 5% sample data.

Fig. S7.

a

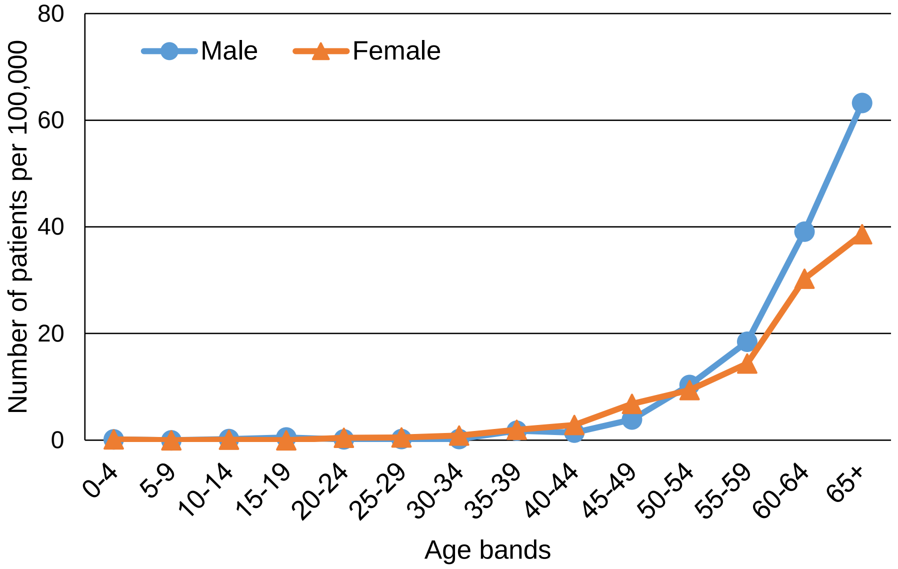

b

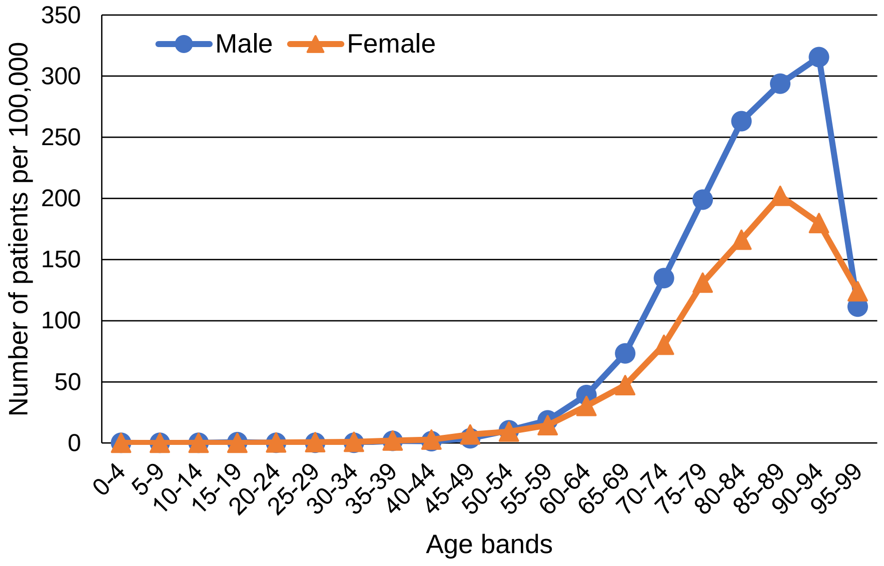

Age- and sex-specific prevalence of idiopathic pulmonary fibrosis in the US using the (a) MarketScan database (January–December 2017) and (b) Medicare database (January–December 2018). MarketScan database, IBM MarketScan Commercial Database; Medicare database, Medicare 5% sample data.

Fig. S8.

a. Male

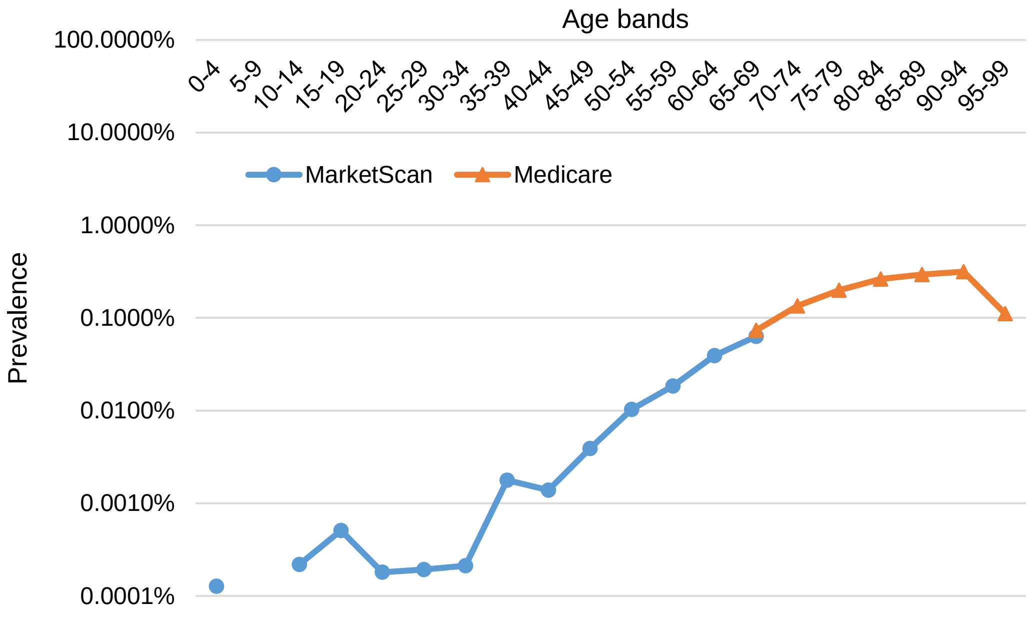

b. Female

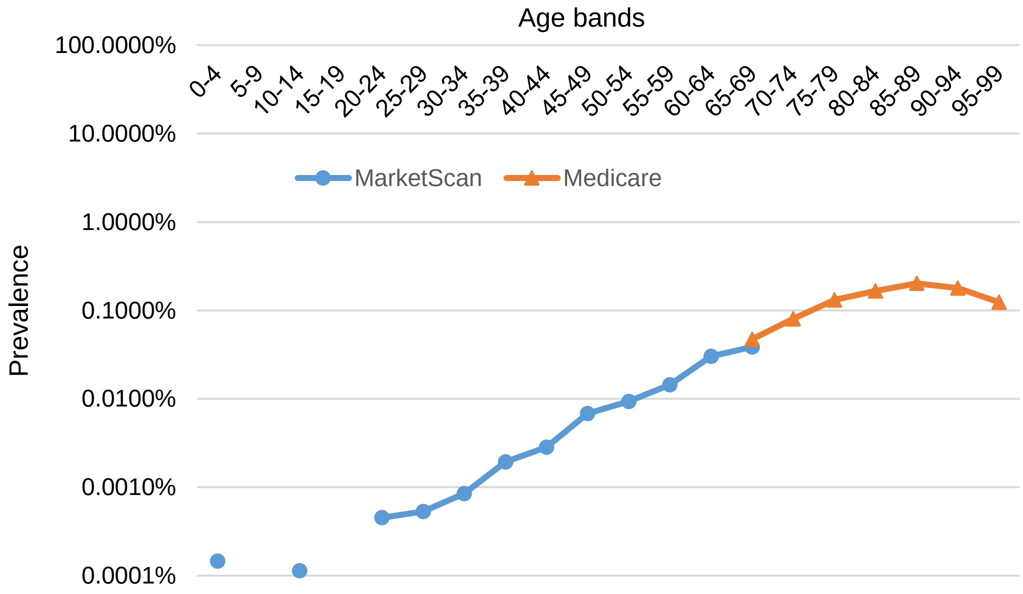

Age- and sex-specific prevalence of idiopathic pulmonary fibrosis in the MarketScan database (<65 years, January–December 2017) and Medicare database (≥65 years, January–December 2018). MarketScan database, IBM MarketScan Commercial Database; Medicare database, Medicare 5% sample data.

Fig. S9.

a. Men

Age	Unknown	White	Black	Other	Asian	Hispanic	North American Native
65-69	103	77	34	64	77	44	191
70-74	80	150	45	120	71	99	0
75-79	134	209	86	260	168	122	423
80-84	0	285	33	359	165	234	239
85-89	515	296	69	530	502	339	510
90-94	0	337	58	775	118	173	1429
95-99	0	109	0	565	391	0	0

b. Women

Age	Unknown	White	Black	Other	Asian	Hispanic	North American Native
65-69	33	51	33	34	22	39	49
70-74	0	85	59	44	76	59	143
75-79	86	145	45	20	65	127	208
80-84	182	176	60	86	191	198	466
85-89	342	218	87	102	66	253	0
90-94	0	189	22	136	235	238	588
95-99	0	132	0	257	0	433	0

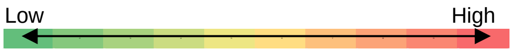

Race-, age- and sex-specific prevalence of idiopathic pulmonary fibrosis in the group aged ≥65 years in the Medicare database (January–December 2018). Prevalence is shown as the number per 100,000 population. Colors are assigned for the number of patients from low to high for each sex as indicated in the color scale bar. Medicare database, Medicare 5% sample data.

Fig. S10.

(A) Men

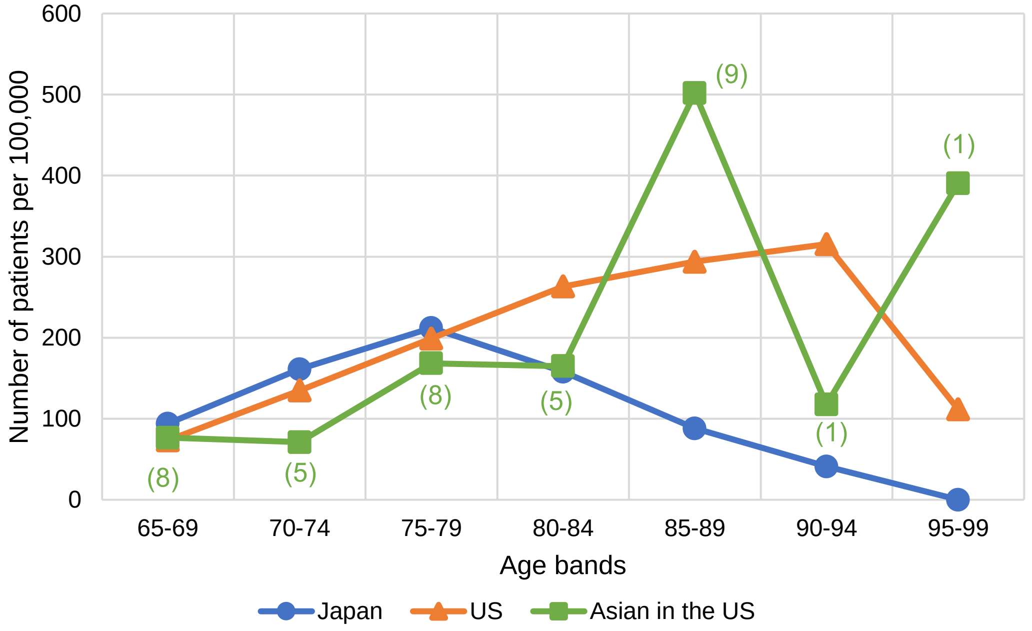

(B) Women

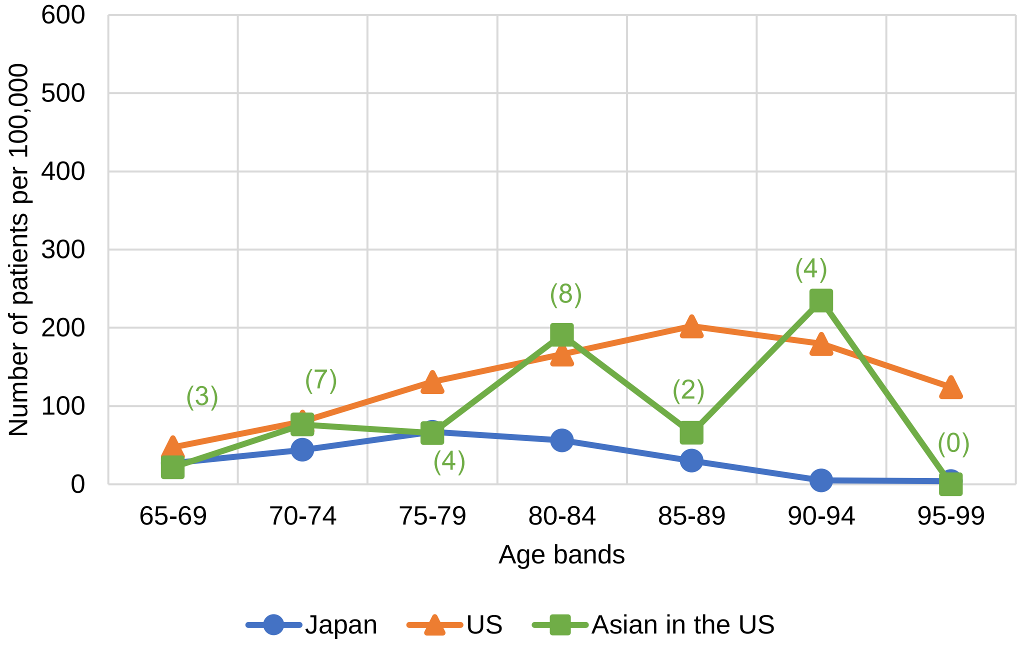

Age- and sex-specific prevalence of IPF in all races and the Asian subgroup in the US and Japan. Numbers in the parentheses represent the number of patients with IPF in Asian subgroup. The prevalence was estimated based on Medical Data Vision Co., Ltd database, fourth NDB Open Data], and demographic data (April 2017–March 2018) for Japan, and Medicare 5% sample data (January–December 2018 for the US, respectively. IPF, idiopathic pulmonary fibrosis.
